# Characterization of distinct polycystic ovary syndrome subtypes by cluster and principal component analyses

**DOI:** 10.3389/fendo.2025.1572427

**Published:** 2025-10-17

**Authors:** Kharis A. Burns, Alexander W. Stuckey, Scott G. Wilson, Gerald F. Watts, Bronwyn G. A. Stuckey

**Affiliations:** ^1^ Medical School, University of Western Australia, Perth, WA, Australia; ^2^ Department of Endocrinology and Diabetes, Royal Perth Hospital, Perth, WA, Australia; ^3^ Department of Endocrinology and Diabetes, Sir Charles Gairdner Hospital, Nedlands, WA, Australia; ^4^ School of Agriculture and Environment, University of Western Australia, Perth, WA, Australia; ^5^ Pink Lake Analytics, Nedlands, WA, Australia; ^6^ School of Biomedical Sciences, University of Western Australia, Perth, WA, Australia; ^7^ Department of Twin Research and Genetic Epidemiology, King’s College London, London, United Kingdom; ^8^ Lipid Disorders Clinic, Department of Cardiology, Royal Perth Hospital, Perth, WA, Australia; ^9^ Keogh Institute for Medical Research, Nedlands, WA, Australia

**Keywords:** polycystic ovary syndrome, PCOS subtypes, body mass index, insulin resistance, metabolic, principal component analysis, cluster analysis

## Abstract

**Introduction:**

Polycystic ovary syndrome (PCOS) is a common, but clinically heterogeneous, condition. This study explores PCOS subtypes using two orthogonal statistical analyses of biochemical and anthropometric data.

**Methods:**

Unsupervised hierarchical cluster analysis and principal component analysis (PCA) of hormonal and metabolic parameters were performed in a cohort of PCOS-affected women, diagnosed based on the NIH criteria. Data collected included body mass index (BMI), blood pressure (BP), fasting insulin and glucose (HOMA-IR), gonadotropins, androgens, and lipids. Subtypes were explored using unsupervised hierarchical cluster analysis, grouping both phenotypic variables and patients into clusters. PCA resolved correlated variables (excluding BMI) into independent factors, and the influence of BMI on the components was then explored.

**Results:**

One thousand and thirty-five women with PCOS were included in the study, with 975 assessed using cluster analysis and PCA. Two main clusters of variables were evident: one characterized by BP, BMI, HOMA-IR, and lipids (triglycerides/cholesterol/LDL) and the second by LH: FSH, androgens, SHBG, and HDL. Three separate patient clusters emerged: cluster A (29.6% of women) showed higher BP, BMI, HOMA-IR, and lipids (triglycerides/cholesterol/LDL) and lower LH: FSH, SHBG, and HDL. Cluster C (43.3%) showed lower BP, BMI, HOMA-IR, triglycerides, testosterone, and FAI and higher LH: FSH, DHEAS, androstenedione, 17-hydroxyprogesterone, SHBG, and HDL. Cluster B (27.1%) was intermediate. Two components aligned with the cluster analysis: principal component (PC) 1, including HOMA-IR, systolic and diastolic BP, triglycerides, LDL, FAI, and SHBG, was positively correlated with BMI (*R* ^2^= 0.32, *p*-value < 0.0001) and aligned with cluster A. PC2, influenced by testosterone, LH: FSH, FAI, DHEAS, androstenedione, and 17-hydroxyprogesterone, with loadings in the opposite direction from LDL and cholesterol, aligned with cluster C, with little relationship with BMI (*R* ^2^= 0.0067, *p*-value = 0.0107).

**Discussion:**

Different metabolic and reproductive PCOS subtypes are evident. Androstenedione and 17-hydroxyprogesterone are important in the reproductive phenotype, highlighting the importance of these hormones in diagnosis and subtype identification and emphasizing their significance in understanding PCOS biology as a predominantly hyperandrogenic disorder. BMI influences and exacerbates the metabolic subtype; in the reproductive group and in lean/normal BMI patients, there is little relationship between weight and other PCOS-related characteristics. Accordingly, traditional treatment paradigms cannot be generalized to all women, and these subtypes may ultimately be viewed as separate disorders

## 1 Introduction

Despite the cardinal features of hyperandrogenism and oligomenorrhea included in the diagnostic criteria for polycystic ovary syndrome (PCOS), there is significant clinical heterogeneity. It is apparent that certain phenotypic traits cluster together, supporting the theory of distinct PCOS subtypes (1). Previous research, including either principal component analysis (PCA) or cluster analysis, has demonstrated that PCOS features cluster together, suggestive of reproductive and metabolic subtypes, contrasting in body mass index (BMI), insulin resistance, and hormone imbalances (1–4). These phenotypes may have different pathophysiology.

Most PCOS-affected women are overweight/obese, with only 16%–30% in the healthy or lean BMI range (5–7), compared with approximately 61% of the general female population in Australia (8). Weight loss and lifestyle changes have been proposed as first-line recommendations to improve menstrual regularity, infertility, hyperandrogenism, and metabolic parameters (9–15).

BMI appears to be a distinguishing trait in terms of the clinical presentation of PCOS. The relative influence of BMI on other PCOS traits and further evidence of clustering of signs/symptoms will add knowledge and direct appropriate management. This study aimed to investigate the relationships between biochemical, hormonal, and metabolic traits in women with PCOS; we hypothesized that phenotypic characteristics were differentially associated with BMI. Characterization of subtypes was explored using both cluster and principal component analyses to provide a wider perspective by comparing two differing approaches and to further elucidate the relationship between phenotype and BMI. The inclusion of a broader androgen and sex steroid profile, including both androstenedione and 17-hydroxyprogesterone, which have not been examined together in other similar studies, aimed to provide additional perspective on PCOS etiology.

## 2 Materials and methods

### 2.1 Study design

First, for the primary analysis, we performed an unsupervised hierarchical cluster analysis of hormonal and metabolic parameters in women with PCOS. Secondly, PCA was performed to identify principal components of linear combinations of correlated phenotypic variables, specifically excluding BMI. Thirdly, the principal components were plotted against BMI to assess correlation.

### 2.2 Study population

Women with PCOS, defined by the National Institute of Health (NIH) criteria (16), were identified through review of the medical records from endocrinology clinics in Western Australia from 1994 until 2018. The diagnosis of PCOS was confirmed by two endocrinologists independently.

Institutional review board ethics approval was obtained from the Department of Health WA Human Research Ethics Committee for Sir Charles Gairdner Hospital and the WA Department of Health (Project Reference Number RGS0000001467). There was reciprocal recognition of ethics approval by the University of Western Australia (reference 2023/ET000911). Due to the size of the cohort and the retrospective nature of data collection over many years, a waiver of patient consent was granted with ethics approval.

### 2.3 Hormone and phenotype data—inclusion/exclusion criteria

Biochemical and phenotype data for each subject from the follicular menstrual phase—luteinizing hormone (LH), follicle-stimulating hormone (FSH), testosterone, SHBG, free androgen index (FAI), dehydroepiandrosterone (DHEAS), androstenedione, and 17-hydroxyprogesterone—were included. Post-menopausal women were excluded from gonadotropin analysis, with diagnosis based on history of oligomenorrhea, clinical features, and/or biochemical evidence of androgen excess. Women taking hormone replacement therapy or the oral contraceptive pill were excluded altogether.

BMI was calculated as weight (kg)/(height (m)^2^. Elevated LH: FSH ratio was defined as ≥ 2. FAI was calculated as (testosterone (nmol/L) × SHBG (nmol/L))/100 (17). There are multiple pathology providers in Western Australia, and consequently, hormone analysis for the patients included in this study occurred across all of these laboratories. Due to differences in the assays used and laboratory standards for calibration, hormone reference ranges for each laboratory vary, particularly regarding androgen analysis. Therefore, direct comparison of androgen levels (including testosterone, SHBG, FAI, DHEAS, androstenedione, and 17-hydroxyprogesterone) could not be performed. Accordingly, the specific assays used at various time points corresponding to the date ranges of samples collected were recorded, and individual reference ranges for each assay and laboratory were obtained and verified. Where applicable, age-specific reference ranges were used, and median data were applied accordingly. Median data were then used to calculate multiples of the median (MOMs), allowing comparison of values across assays (18).

Insulin resistance was expressed using the homeostatic model assessment of insulin resistance (HOMA-IR) (19–21). Based on a model developed using local population data, ≥1.8 was defined as insulin resistance (7). Lipid thresholds—cholesterol >5.5 mmol/L, triglycerides >1.7 mmol/L, low-density lipoprotein (LDL) >3.0 mmol/L, and high-density lipoprotein (HDL) <1.0 mmol/L—were derived from the WHO definition of metabolic syndrome (22).

### 2.4 Statistics

SAS software version 9.4 and R version 4.2.2 were used for initial analyses. Means (standard deviations) and medians (quartiles (Q1, Q3)) were used for normally and non-normally distributed variables, respectively. R version 4.1.2 was used for the cluster and principal component analyses.

### 2.5 Missing variables

There was some missingness at the item level in the PCOS dataset. The joint missingness across the variables is displayed in the upset plot in **Supplementary Figure S1**. In general, there tended to be a group of metabolic variables missing or a group of reproductive variables missing. For principal component analysis and clustering, missing values were imputed. The imputation method was as follows: records with only BMI, records with only BMI and blood pressure, and records containing only BMI, testosterone, and SHBG were removed; for each remaining record with missing data, imputation was performed for missing variables by using the clustering and imputation algorithm given in the ClustImpute R package (23). The number of clusters to be used by the ClustImpute algorithm was set at 3. We here report a single run of the algorithm as a whole, using the software’s default seed.

Principal component loadings were calculated for the imputed dataset and the set of complete cases. The comparison in **Supplementary Figure S2** shows that there was little difference between these results for the two datasets.

### 2.6 Cluster analysis

Agglomerated unsupervised hierarchical cluster analysis based on Ward’s minimum variance method on Manhattan distances between trait values was performed (24, 25). This method explores relationships between traits, without applying assumptions, to identify subgroups of patients with similar phenotypical characteristics (24, 25). The method implemented follows that of a previous study, including inverse normal transformation of the variables (2). Fifteen variables were included: systolic and diastolic blood pressure (SBP and DBP), BMI, LH: FSH, HOMA-IR, testosterone, SHBG, FAI, DHEAS, androstenedione, 17-hydroxyprogesterone, cholesterol, triglycerides, LDL, and HDL.

### 2.7 Principal component analysis

The same 15 variables, excluding BMI, were incorporated in the PCA. The method used for this analysis was the “prcomp” function from the built-in “stats” R package (26). This approach groups related variables into distinct statistically independent orthogonal components. The first component explains the highest proportion of the variance in the traits, the second the next highest proportion of variance, and so on. Subsequently, the relationship between BMI and the principal components identified was examined separately.

This approach was utilized to explore whether groups of variables may be distinct to support the concept of subtypes. The exclusion of BMI was intended to examine these subtypes without using BMI as a key component of PCOS. This novel methodological approach examined the relationship between BMI and the components separately and then aimed to see how BMI interacts with the PCOS phenotypes.

## 3 Results

### 3.1 Baseline data

The study population comprised 1,035 women; baseline descriptive data are presented in **Table 1**. The median age of the subjects was 28 (22.5, 34.6) years, 28% (*n* = 290) had lean/normal BMI, 30.4% displayed elevated LH: FSH (≥2), and most women had SHBG levels below assay medians [median SHBG (MOM) 0.5 (Q1, Q3 0.4, 0.6)]. The median MOMs for DHEAS, androstenedione, and 17-hydroxyprogesterone were >1, indicating that most women had these steroids above the respective assay medians.

**Table 1:** Baseline descriptive data of PCOS cases.

Data	Median	IQR
Age at diagnosis (years)	28	22.5, 34.6
BMI (kg/m^2^)	30.1	24.6, 36.6
LH: FSH <2	1.0	0.7, 1.4
LH: FSH ≥2	2.7	2.2, 3.3
HOMA-IR	2.7	1.5, 4.6
Diabetes^a^, *n* (%)	20	2.75
Total cholesterol (mmol/L)	4.8	4.2, 5.6
Triglycerides (mmol/L)	1.1	0.8, 1.6
LDL (mmol/L)	3.0	2.4, 3.6
HDL (mmol/L)	1.2	1, 1.5
Testosterone	1.8	1.3, 2.5
SHBG	0.5	0.3, 0.8
Free androgen index	4	2.1, 6.8
DHEAS	1.2	0.8, 1.6
Androstenedione	1.5	1, 2
17-Hydroxyprogesterone	1.6 (1, 2.5)	1, 2.5
SBP (mmHg)	112.5 (105, 125)	105, 125
DBP (mmHg)	70 (60, 75)	60, 75

a Diabetes refers to the proportion of patients who had diabetes at the time of PCOS diagnosis, based on fasting glucose or OGTT results. HOMA_IR was calculated by (fasting plasma glucose × fasting insulin)/22.5. HOMA-IR *n* values are lower than fasting insulin and glucose levels, as there were some patients with fasting insulin or fasting glucose results (i.e., not collected at the same time). Free androgen index was calculated by (testosterone × SHBG)/100. All parameters presented as median (Q1, Q3), except where specified. Testosterone, SHBG, free androgen index, DHEAS, androstenedione, and 17-hydroxyprogesterone all presented as MOMs (multiples of the median).

Over two-thirds (67.5%) of women, with available data, displayed insulin resistance (HOMA-IR ≥1.8), and the median HOMA-IR was 2.7. Diabetes was evident at diagnosis in 20 patients (2.75%) according to fasting (≥7.0 mmol/L) and/or 2-h (>11.0 mmol/L) glucose levels.

Nine hundred and seventy-five women were included in the cluster and principal component analyses after following the imputation process.

### 3.2 Hierarchical cluster analysis

**Figure 1:**
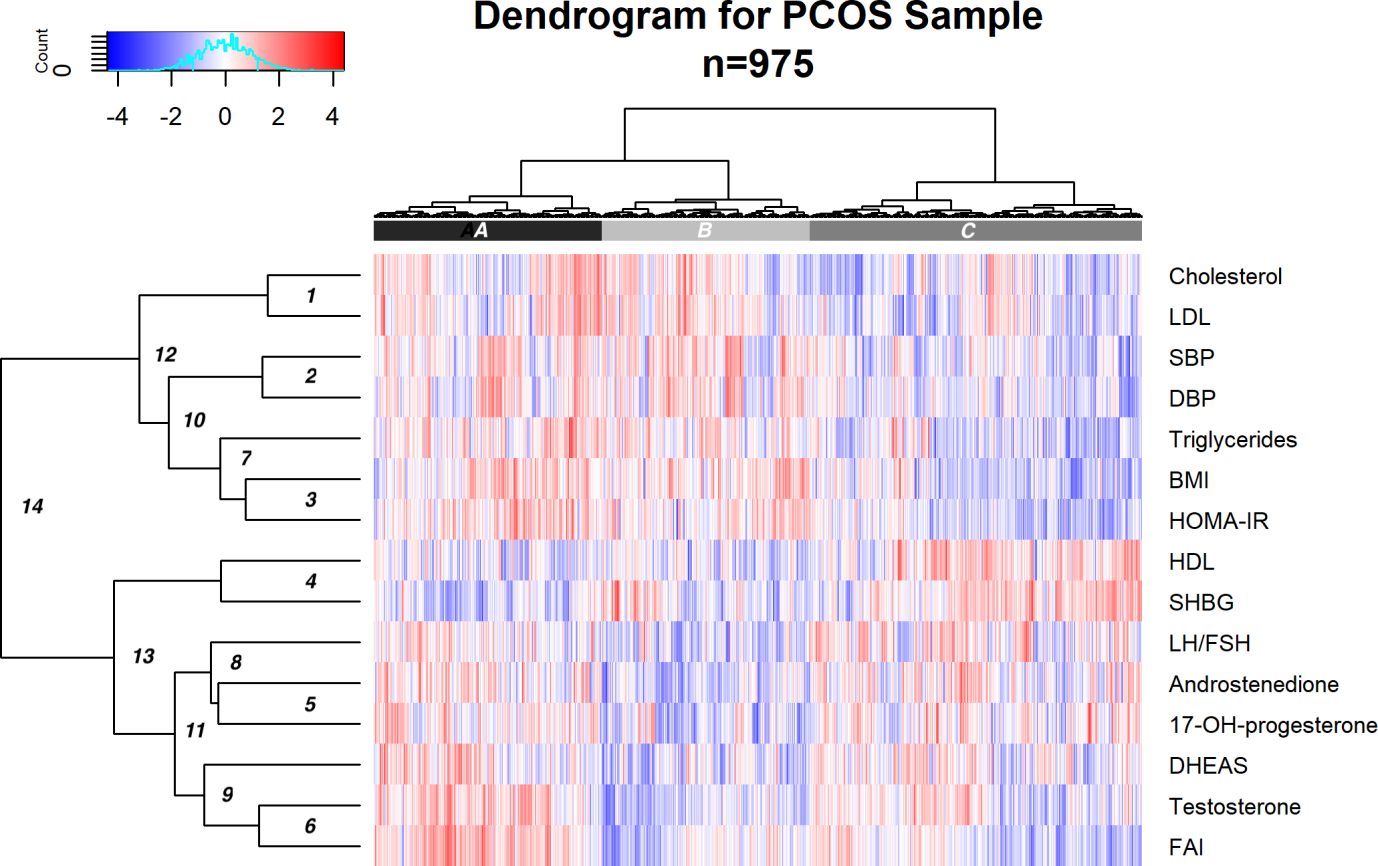
Dendrogram illustrating agglomerated hierarchical clustering within the WA PCOS cohort (*n* = 975). This is designed to be interpreted left to right to show three clusters of patients and top to bottom across rows to look at clustering of PCOS-related traits. The key describing the color scale is in the left-hand upper corner. Colors represent *z*-scores for each of the PCOS-related traits; shades of red and blue correspond to high and low values, respectively. The vertical axis demonstrates the similarity or dissimilarity between traits or clusters. More similar traits are clustered together, connected by shorter branches. The order of the rows is arranged so that when two clusters of variables are joined, the tighter of the two clusters is ordered above. The rows represent each of the 15 PCOS traits included in the cluster analysis. Each vertical line within that row represents a single patient result. Each vertical column represents a single patient across the different variables included. SBP, systolic blood pressure; DBP, diastolic blood pressure; BMI, body mass index; HOMA-IR, homeostatic model of insulin resistance; triglycerides; testosterone (MOM); FAI, free androgen index (MOM); LH: FSH, luteinizing hormone: follicle-stimulating hormone; 17-hydroxyprogesterone (MOM); DHEAS, dehydroepiandrosterone (MOM); androstenedione (MOM); cholesterol; LDL, low-density lipoprotein; HDL, high-density lipoprotein; SHBG, sex hormone-binding globulin (MOM). The horizontal bar at the superior margin of the diagram shows three shaded areas—(A) black, (B) gray, and (C) light gray. This shows separation in three PCOS phenotypes: (A) 29.6% of the sample, most consistent with the metabolic phenotype with higher BMI; (B), 27.1%, an intermediate group; and (C) 43.3%, a reproductive phenotype with lower BMI.

The dendrogram in **Figure 1** illustrates the hierarchical clustering of patients and variables. The clustering is agglomerative, in that patients that are more similar with respect to the variables are paired first and then increasingly large clusters are formed until there is one single cluster (24). The earlier in the agglomerative process that two patients are joined in the same cluster, the more similar they are to each other. When two clusters are joined, the tighter of the two clusters is presented to the left.

It should be noted that some of the variables used in the cluster analysis are related in some direct way, which may increase their influence on the clustering of individuals. For example, FAI is derived from testosterone and SHBG. This interaction is of interest because of the relative difference in the driver of androgen excess in each subtype (primary androgen excess versus low SHBG). The pairwise correlations between the variables are displayed in a heatmap in **Supplementary Figure S3**.

#### 3.2.1 Clustering of phenotypic traits (vertical axis)

Initial clusters (1–6 on the dendrogram, **Figure 1**) showed relationships between the following pairs: 1) cholesterol and LDL, 2) SBP and DBP, 3) BMI and HOMA-IR, 4) HDL and SHBG, 5) androstenedione and 17-OH-progesterone, and 6) testosterone and FAI. At this initial level, triglycerides and LH: FSH did not pair with any other variable. At the next level, triglycerides join cluster 7, containing HOMA-IR and BMI. LH: FSH combines with androstenedione and 17-OH-progesterone to form cluster 8, and DHEAS joins testosterone and FAI to form cluster 9.

Beyond, two clusters were apparent: SBP, DBP, triglycerides, BMI, and HOMA-IR (cluster 10) and LH: FSH, androstenedione, 17-hydroxyprogesterone, DHEAS, testosterone, and FAI (cluster 11). Cholesterol and LDL then joined cluster 10 (cluster 12), and HDL and SHBG joined cluster 11. Finally, two overall subtypes were evident: cluster 12 characterized by cholesterol, LDL, blood pressure, BMI, HOMA-IR, and triglycerides, which segregated from cluster 13, containing HDL, SHBG, LH: FSH, androstenedione, 17-OH progesterone, DHEAS, testosterone, and FAI. These final two clusters merge to form PCOS overall. The metabolic parameters, SBP, DBP, HOMA-IR, BMI, and triglycerides, display stronger correlation than those in the other group, including LH: FSH and androgens.

#### 3.2.2 Patient clustering (horizontal axis)

Three clusters of patients were apparent: A, 29.6%; B, 27.1%; and C, 43.3% of the sample, respectively (**Figure 1**). Cluster A patients had higher cholesterol, LDL, BP, BMI, HOMA-IR, and triglycerides and lower LH: FSH, SHBG, and HDL. In contrast, cluster C patients had lower cholesterol, LDL, SBP and DBP, BMI, HOMA-IR, triglycerides, testosterone, and FAI and higher LH: FSH, DHEAS, androstenedione, 17-OH-progesterone, SHBG, and HDL. The middle cluster (B) showed indeterminate patterns in the measures.

Each of the variables was compared between clusters A and C, with evidence of significant differences in HOMA-IR, FAI, BMI, triglycerides, LDL, cholesterol, SBP, DBP, testosterone, HDL, and SHBG between groups. Significant differences in means are annotated with an asterisk, according to a two-sample *t*-test with Bonferroni-corrected *p*-values of less than 0.05 (**Supplementary Figure S4**).

**Figure 2:**
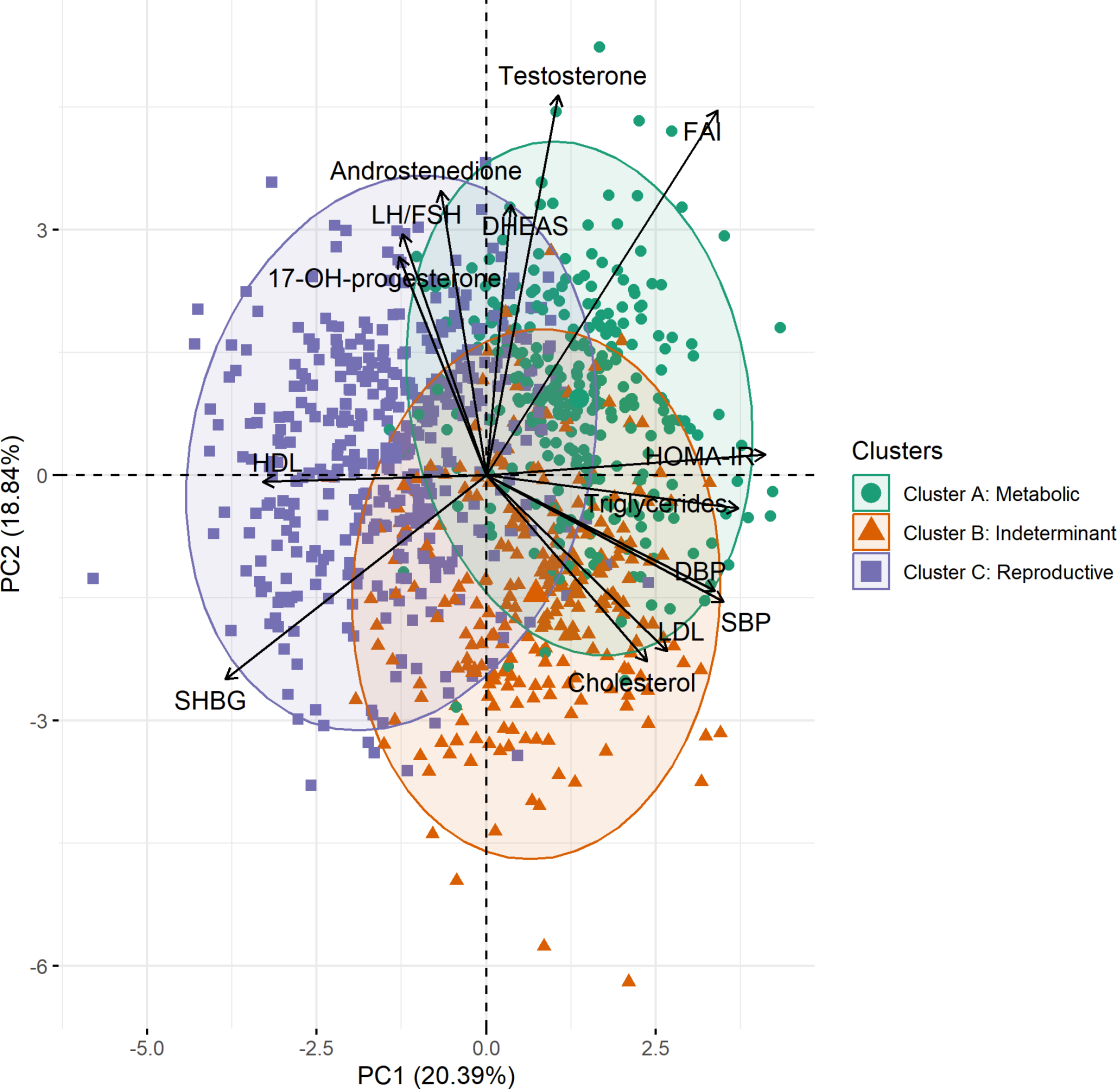
Plot generated from principal component analysis combined with the results from cluster analysis. Each case (represented by a circle, triangle, or square) is plotted in the color relative to its cluster subtype [cluster A, green (metabolic); cluster B, red (indeterminant); and cluster C, purple (reproductive)]. The first two principal components (PC1 and PC2) are displayed orthogonally on the *x*- and *y*-axes, respectively. The black arrows represent the direction and magnitude of each of the variables included in the principal component analysis. The metabolic subtype shows distinct separation from the reproductive, most evident with clustering patterns around insulin resistance (HOMA-IR), which is closely aligned with the first principal component. PC, principal component; HOMA-IR, homeostatic model of insulin resistance; HDL, high-density lipoprotein; LDL, low-density lipoprotein; LH, luteinizing hormone; FSH, follicle-stimulating hormone; SHBG, sex hormone-binding globulin; FAI, free androgen index; DHEAS, dehydroepiandrosterone; AND, androstenedione; 17-OHP, 17-hydroxyprogesterone; SBP, systolic blood pressure; DBP, diastolic blood pressure.

Jaccard scores were calculated using bootstrap resampling. For clusters A, B, and C, the scores were 0.47, 0.51, and 0.61, respectively. While the score for cluster C shows good stability, the scores for clusters B and A indicate that the groups are overlapping in structure, and individuals can switch groups in bootstrap samples. These characteristics can be seen visually in the overlapping clusters of individuals when plotted against the first two principal components in **Figure 2**. Running 1,000 simulations of the ClustImpute algorithm on the dataset yielded an average adjusted Rand index of 0.463.

### 3.3 Principal component analysis

The first four principal components (PC1–PC4) were relevant to explain the variability in the sample using a scree plot and accounted for 58.54% of the variance in the cohort (**Table 2**). The first principal component (PC1), accounting for 20.39% of the variance, contained HOMA-IR, triglycerides, SBP, DBP, FAI, cholesterol, and LDL with positive coefficients and SHBG and HDL with negative coefficients as the dominant variables.

**Table 2:** Principal component analysis results and coefficients.

Variable	PC1	PC2	PC3	PC4
HOMA-IR	0.39	0.01	−0.11	−0.22
SHBG	−0.38	−0.24	0.26	0.19
Triglycerides	0.36	−0.05	0.21	−0.14
FAI	0.34	0.43	−0.10	−0.08
SBP	0.33	−0.15	−0.03	0.61
HDL	−0.32	−1.00E−04	0.23	0.20
DBP	0.30	−0.14	−0.03	0.61
LDL	0.26	−0.22	0.46	−0.20
Cholesterol	0.23	−0.23	0.58	−0.12
17-OH-Progesterone	−0.12	0.27	0.32	0.03
LH/FSH	−0.12	0.31	0.25	0.12
Testosterone	0.11	0.46	0.13	0.12
Androstenedione	−0.06	0.35	0.28	0.13
DHEAS	0.04	0.33	0.07	0.10
Percentage of variance (%)	20.39	18.84	10.54	8.77

PC, principal component; HOMA-IR, homeostatic model of insulin resistance; HDL, high-density lipoprotein; LDL, low-density lipoprotein; LH, luteinizing hormone; FSH, follicle-stimulating hormone; SHBG, sex hormone-binding globulin; FAI, free androgen index; DHEAS, dehydroepiandrosterone; SBP, systolic blood pressure; DBP, diastolic blood pressure.

### 3.4 Relationships between BMI and principal components

Plotting PC1 against the normalized BMI score for each patient (**Supplementary Figure S5**) demonstrates a linear relationship (*R* ^2^ = 0.32, *p*-value < 0.0001); thus, 32% of the variance in BMI is explained by PC1, which was derived without reference to BMI.

PC2 is most strongly influenced by FAI, testosterone, DHEAS, 17-hydroxyprogesterone, LH: FSH, and androstenedione in a similar direction, with loadings in the opposite direction from SHBG, LDL, and cholesterol. PC2 accounts for 18.84% of the variance in the sample and showed a very weak association with BMI (*R* ^2^ = 0.0067, *p*-value 0.0107) (**Supplementary Figure S6**).

Cholesterol, LDL, LH: FSH, androstenedione, and 17-hydroxyprogesterone carry the most weight in PC3. PC3 shares some similarities with PC2, with LH: FSH, androstenedione, and 17-hydroxyprogesterone demonstrating greater contribution. PC4 is influenced by LDL in the opposite direction to SBP and DBP. PC3 and PC4 also show a lack of relationship with BMI (*R* ^2^ = 0.0374 and 0.0025, respectively).

## 4 Discussion

### 4.1 Key findings

This study has demonstrated the relationship of obesity to the broad and heterogeneous PCOS clinical phenotype. The cluster analysis revealed the place of BMI in the hierarchy of PCOS phenotypic features. The deliberate and novel exclusion of BMI from the PCA demonstrated principal components within PCOS autonomous from BMI, allowing analysis of the relationship between these components and BMI without prior assumption.

This approach extends the understanding of PCOS subtypes and clarifies the relationships between these and BMI. The metabolic subgroup is predominantly driven by insulin resistance and exacerbated by obesity. In contrast, the reproductive phenotype is characterized by hyperandrogenism and elevations in sex steroids, with body weight having a negligible role in the pathophysiology. Such distinctions are important, not only for identifying and understanding pathophysiology but also for planning targeted therapy.

The findings from this research expand upon the initial PCA conducted by Stuckey et al. (1) and on the more recent cluster analysis findings in the literature (2–4) by using a broader range of sex steroids, including androstenedione and 17-hydroxyprogesterone, not previously included (1–4).

### 4.2 Cohort characteristics

Our cohort’s baseline characteristics are similar to other cohorts in the literature. Most women were overweight (5, 6), and most had SHBG levels below the median, with lower levels in overweight/obese BMI groups recognized (27, 28). Insulin resistance was common, with a median HOMA-IR of 2.7 (1.5, 4.6) as is reported in other studies in Caucasian populations (7, 29–31). Notwithstanding this cohort’s similarity to others reported in the literature, and despite the use of the NIH criteria, rather than the much broader Rotterdam criteria, distinct subtypes are evident within the umbrella diagnosis of PCOS.

### 4.3 Cluster and principal component analyses

Early combinations of variables within the phenotype cluster analysis (**Figure 1**) found expected concordance between insulin resistance and hypertriglyceridemia, consistent with the known anabolic actions of insulin and regulation of lipid metabolism, with inhibition of lipolysis (32). The early combination of BMI with HOMA-IR and then triglycerides demonstrates the contribution of intrinsic insulin resistance as a primary contributor in this PCOS cluster (33).

The “metabolic” cluster emerges with the next addition of SBP and DBP (cluster 10) and is ultimately clear in cluster 12. Patient cluster A shares the same phenotypic variables as phenotypic cluster 12, identifying a “metabolic” PCOS phenotype. A similar pattern was identified through PCA. PC1, also dominated by metabolic variables, explained the largest proportion of the variance in the cohort. The opposing directions of the variables in PC1, with HOMA-IR positive and SHBG negative, align with clinically observed relationships between these features of PCOS. Together, these orthogonal statistical approaches are concordant in demonstrating segregation of traits concerning metabolic risk in patients with a metabolic phenotype. The deliberate omission of BMI from the PCA, and the subsequent relationship identified by plotting PC1 against BMI, supports the notion that insulin resistance, not BMI, is the initial driving variable in this group.

In contrast, the clustering of gonadotropin ratios, androgens, and sex steroids provides evidence of another distinct PCOS phenotype. Early union of high LH: FSH, androstenedione, and 17-hydroxyprogesterone (cluster 8, **Figure 1**), with further agglomeration with DHEAS, testosterone, and FAI (cluster 11), demonstrates clear separation from cluster 10. Collectively, these parameters are often seen in women with PCOS, who are of lean or normal body weight (34) and could be considered as the “reproductive phenotype,” a term coined by other research groups and demonstrated here (2, 4, 35). This group could also be viewed as an “adrenal phenotype” with a subtle contribution from adrenal androgens. Because both androstenedione and 17-hydroxyprogesterone may be of ovarian origin, the addition of 21-deoxycortisol and other exclusive adrenal androgens to future hormonal investigation panels may help clarify the adrenal contribution to the etiology of this PCOS phenotype (36, 37). These hormones may also indicate other pathologies, such as non-classic CAH, with overlapping or shared features of hyperandrogenism seen in this phenotype.

The second principal component (PC2) supported the cluster analysis findings, showing greater contribution from testosterone, FAI, LH: FSH, androstenedione, DHEAS, testosterone, and 17-hydroxyprogesterone. This component supports the analysis by our group and others (1–4), although the additional approach to investigate the relationship between clinical features and body weight differs in the present study.

The concordance of the cluster analysis findings and the first two principal components (**Figure 2**) upholds the subtypes identified in each. Insulin resistance is orthogonal to gonadotropin imbalance (LH: FSH) and the adrenal androgens. The intermediate or shared position of FAI, between PC1 and PC2, is unsurprising given that hyperandrogenism is an essential criterion in the NIH diagnostic criteria (16). The positions of SHBG and testosterone, relative to PC1 and PC2, demonstrate the contribution of each to hyperandrogenism. In the metabolic subtype, hyperandrogenemia results from low SHBG and in the reproductive subtype primarily from elevated testosterone (38, 39).

#### 4.3.1 The influence of broader androgens and sex steroids: a possible adrenal contribution

The inclusion of both androstenedione and 17-hydroxyprogesterone in this study differs from previous analyses (2–4, 35). The early union of 17-hydroxyprogesterone with LH: FSH in the cluster analysis suggests that the PCOS phenotype may be driven by variants in genes controlling the adrenal enzyme pathway, a pathology referred to as prenatal “androgenization of the hypothalamic pituitary ovarian axis” (40). Although 17-hydroxyprogesterone should be measured to distinguish PCOS from congenital adrenal hyperplasia (CAH) (41), there is some overlap in basal 17-hydroxyprogesterone between PCOS and non-classical and heterozygote CAH (42). Other analytes, including 21-deoxycortisol, have been reported to help discriminate forms of CAH from PCOS (36).

Androstenedione has been considered of little diagnostic or clinical utility in PCOS (43–45), though the findings herein dispute that view. Androstenedione was significantly higher in PCOS-affected women compared to controls in other research, though notable differences in levels between BMI subgroups were not seen in that study (34). In adolescents with PCOS, increased androstenedione, LH, and testosterone have been reported in lean girls with PCOS (46).

Our research has previously demonstrated significantly higher androstenedione levels in lean women compared with overweight and obese PCOS BMI strata (**Supplementary Table S1**) (47). Furthermore, the results presented herein show that in PC2, where BMI has a negligible role, androstenedione is an important component, as demonstrated by the weighting in the principal component analysis, and is therefore an informative analyte. However, androstenedione, despite a loading in PC2 but not PC1, was not informative in categorizing individuals into cluster analysis subtypes (**Figure 2**).

Although the source of hyperandrogenism in PCOS is primarily ovarian, the adrenal glands are also implicated in androgen production (48). The relative contribution of ovarian and adrenal androgen production may vary, however, between different PCOS subtypes, reflecting the condition’s heterogeneity. The exact mechanisms behind adrenal androgen excess in PCOS are yet to be elucidated, with proposed pathophysiologies including altered adrenal steroidogenesis enzyme activity and/or alternative androgen synthesis pathways (49, 50). Although PCOS is considered distinct from late-onset congenital adrenal hyperplasia, some women with PCOS demonstrate more resemblance to such primary adrenal disorders than to those women with metabolically driven PCOS. Elevated levels of androstenedione, in particular, may signal a stronger adrenal influence on androgen production in subtype populations.

The use of unsupervised hierarchical cluster analysis without assumption and the deliberate exclusion of BMI from the PCA sought to explore the role of obesity in the PCOS phenotype. The cluster analysis identified insulin resistance but not BMI in the first cluster of phenotypic variables. Similarly, PC1 was identifiable without the inclusion of BMI in the PCA. Thereafter, PC1 was found to be correlated with BMI, with only a very weak correlation between BMI and PC2. The pathophysiology of the metabolic PCOS subtype is likely predominantly driven by insulin resistance, exacerbated, but not initiated, by overweight/obesity, and ameliorated by weight loss. It is the group that is most likely to respond positively to dietary and lifestyle therapeutic interventions (51). Conversely, insulin resistance conferred a negligible impact on PC2, nor was PC2 strongly correlated with BMI (**Supplementary Figure S6**). Therefore, this suggests that management in subtypes should have a different initial focus, targeting insulin resistance in those with the metabolic subtype and amelioration of androgen excess in the reproductive subtype.

### 4.4 Clinical significance

These distinctions in phenotype suggest differences in genetics (52), in pathophysiology, and most importantly, in the approach to management. The metabolic subtype, where insulin resistance is the primary driver in PCOS pathophysiology, should respond to weight management and lifestyle measures. In contrast, those that do not fit the metabolic phenotype, rather the reproductive subtype, sharing similarities with conditions such as CAH, demonstrating elevated androstenedione and 17-hydroxyprogesterone (40), are characterized by an interplay between gonadotropin imbalance, predominated by LH, and hyperandrogenism. In these women, although weight gain may exacerbate the clinical features, obesity is not the driving force, and weight loss and lifestyle measures are unlikely to address the PCOS phenotype. Alternative management should be considered. What that management is probably depends upon a clearer understanding of the genetic etiology than we presently have. In the context of available treatment options, the primary focus for lean women or those with the reproductive phenotype should be on mitigating androgen excess. Pharmacotherapy, including the estrogen-containing oral contraceptive pill and anti-androgens such as cyproterone acetate and spironolactone, aimed at mitigating both androgen production and receptor action, is the main option and should be considered early in this subtype. More targeted therapeutic interventions will likely require a greater understanding of the pathophysiology, including the genetic variance, of the respective phenotypes.

The use of a robustly characterized, uniformly diagnosed cohort of PCOS-affected women using the NIH criteria is a strength in this study, in that, despite maximizing the homogeneity of the population, clear evidence of subtypes emerged. The broader range of phenotype data, including DHEAS, androstenedione, and 17-hydroxyprogesterone, highlights the role of these hormones in understanding PCOS subtypes. Other literature to date has not been as extensive in the inclusion of this full range of variables (1–4). The use of PCA excluding BMI is a novel approach that allows the identification of subtypes without the supposition of BMI influence. Despite the large amount of data included in this study, instances of some missing data were a recognized limitation.

The imputation algorithm has a stochastic element, so we acknowledge that repeated runs do not yield the same imputed values. This is a recognized limitation of imputation processes. The Rand index shows that the clustering is not highly stable under repeated application of the imputation algorithm. The fact that some patients do not clearly belong to one cluster is also evident in the Jaccard scores of the clusters and visualized by the overlapping clusters when presented against PC1 and PC2 in **Figure 2**. While not disjoint, the metabolic and reproductive clusters are clearly evident and moderately stable.

Another limitation may be that the data were collected over a long period, and hormone assays differed both over time and between laboratories. Therefore, as discussed above, MOMs were used to facilitate comparison. Variability of gonadotropins was considered less significant given that ratios were of most interest. All samples were collected fasting and excluded the mid-cycle or luteal phase. Post-menopausal women were excluded from hormonal analysis, with hormone data only included if collected prior to menopause. This was a retrospective study; hence, uniformity in assays, while ideal, was not entirely possible.

Future studies should aim to replicate these findings in women of varied ethnicity in larger cohorts, acknowledging the potential differences in PCOS phenotype in women of different backgrounds. The application of this same approach to a group of Rotterdam-defined PCOS patients would also be of interest and likely to identify further subtypes, given the broader diagnostic criteria. Although the Rotterdam criteria are widely accepted for PCOS diagnosis, this study purposely utilized a more strictly defined cohort to maximize homogeneity in the quest to delineate subtypes.

With the increasing prevalence of artificial intelligence in medical practice, the role of such technology could also be evaluated, potentially examining algorithms and techniques to predict PCOS subtype in a clinical setting.

The role of anti-Müllerian hormone in the delineation of the PCOS subtype is of interest; however, due to the recent introduction of this analyte, relative to the longevity of retrospective data collection for this cohort, data were sparsely available. AMH has recently been accepted as a diagnostic tool in PCOS workup, as an alternative to ultrasound, depending on the diagnostic criteria applied (45). It has also been associated with a reproductive phenotype in another cluster analysis, together with elevated SHBG, LH, and antral follicle count (4). The inclusion of this variable in this study design, together with the broader androgen profile adopted, may have further clarified the distinction in subtypes and validated other research, illuminating the role of this hormone in PCOS phenotype identification. It is noted, however, that age complicates the diagnostic utility of AMH, with higher levels noted in younger women (53).

Other variables not included in this research, which are potentially implicated in PCOS phenotype and warrant further exploration, include markers of cardiometabolic risk such as apolipoprotein B (apoB) and remnant cholesterol, adiponectin, and ambulatory blood pressure monitoring. Imaging characteristics such as computerized tomography (CT) and DEXA, investigating liver adiposity and fat distribution, would also add a valuable contribution to understanding PCOS subtypes and relative metabolic risk.

This study and similar other studies raise important questions about the heterogeneity of PCOS. These two statistical methods contribute to the understanding of this heterogeneity, and future research is required to evaluate the contribution of individual metabolites to dissecting the subtypes. The metabolic phenotype is clearly defined. However, further study with more patients, more analytes, and a deeper understanding of adrenal steroid production is necessary to fully characterize those women who do not fit a metabolic phenotype. Although we have identified two groups, the “reproductive” phenotype is still likely an umbrella term awaiting further dissection of the heterogeneity. An evaluation of long-term outcomes in these PCOS subtypes will be essential to expand this understanding.

## 5 Conclusion

We conclude that there are two distinct subgroups of PCOS: a reproductive/androgen-driven group and a metabolic/insulin-driven group. Body weight only associates with features in the metabolic PCOS phenotype, supporting lifestyle measures as first-line treatment in these women. These findings challenge the notion that insulin resistance is the dominant inherent driver in all women with PCOS. This study supports the reclassification of PCOS phenotypes into distinct entities with separate therapeutic approaches.

## Data Availability

The original contributions presented in the study are included in the article/**Supplementary Material**. Further inquiries can be directed to the corresponding author.

## Supplementary material

Supplementary Figure 1 Upset Plot for missing data. This upset plot shows the structure of missingness in the dataset. The most commonly missing variable was 17-OH-progesterone which was missing in over 300 cases. The most common set of variables jointly missing was cholesterol, HDL, LDL and HOMA which were missing together 57 times.

Supplementary Figure 2 Principal component loadings for complete-case dataset and dataset with imputation for missing data. The loadings for the first and second principal component are shown under two scenarios. The complete case scenario omits any records with missing data, leaving 294 complete records. This is compared to the full dataset of 975 with missing values imputed. The plot shows that the principal component loadings are very similar in each case.

Supplementary Figure 3 Heatmap showing pairwise correlations between variables. Each cell represents the Pearson correlation coefficient (r) between variable pairs. Colour intensity, illustrated with the key on the right of the diagram, indicates the strength and direction of the correlation, with red indicating positive correlations and blue indicating negative correlations. Values range from –1 (strong negative correlation, blue) to +1 (strong positive correlation, red).

Supplementary Figure 4 Comparison of trait distributions between metabolic and reproductive clusters. Each of the variables were compared between Clusters A and C with evidence of significant difference in HOMA-IR, FAI, BMI, Triglycerides, LDL, cholesterol, SBP, DBP, testosterone, HDL and SHBG between groups. Significant differences in means are annotated with an asterisk, according to a two-sampled t-test with Bonferroni corrected p-values < 0.05.

Supplementary Figure 5 Scatterplot of the first principal component (PC1) plotted on the X-axis against the normalised BMI score on the Y axis, with degree of correlation R^2^ = 0.3204, p-value <0.0001. The second component (PC2) is most strongly influenced by LH: FSH, testosterone, FAI, DHEAS and androstenedione in a similar direction, with large loadings in the opposite direction from SHBG, LDL and cholesterol. This component accounts for 18.84% of the variance in PCOS. The relationship between BMI and the second component was very weak, suggesting that BMI is less influential in this component (R^2^ = 0.0067,p-value 0.0107) (**Supplementary Figure S6**).

Supplementary Figure 6 Scatterplot of the second principal component (PC2) plotted on the X-axis against the normalised BMI score on the Y axis.Relationship between PC2 and BMI is negligible, with only very weak association demonstrated R^2^ = 0.0067, p-value 0.0107.
