# Supplementary figures and images for: Characterization of distinct polycystic ovary syndrome subtypes by cluster and principal component analyses
Source: Front Endocrinol (Lausanne). 2025 Oct 17;16:1572427. doi: 10.3389/fendo.2025.1572427 (PMC12575186; doi:10.3389/fendo.2025.1572427)

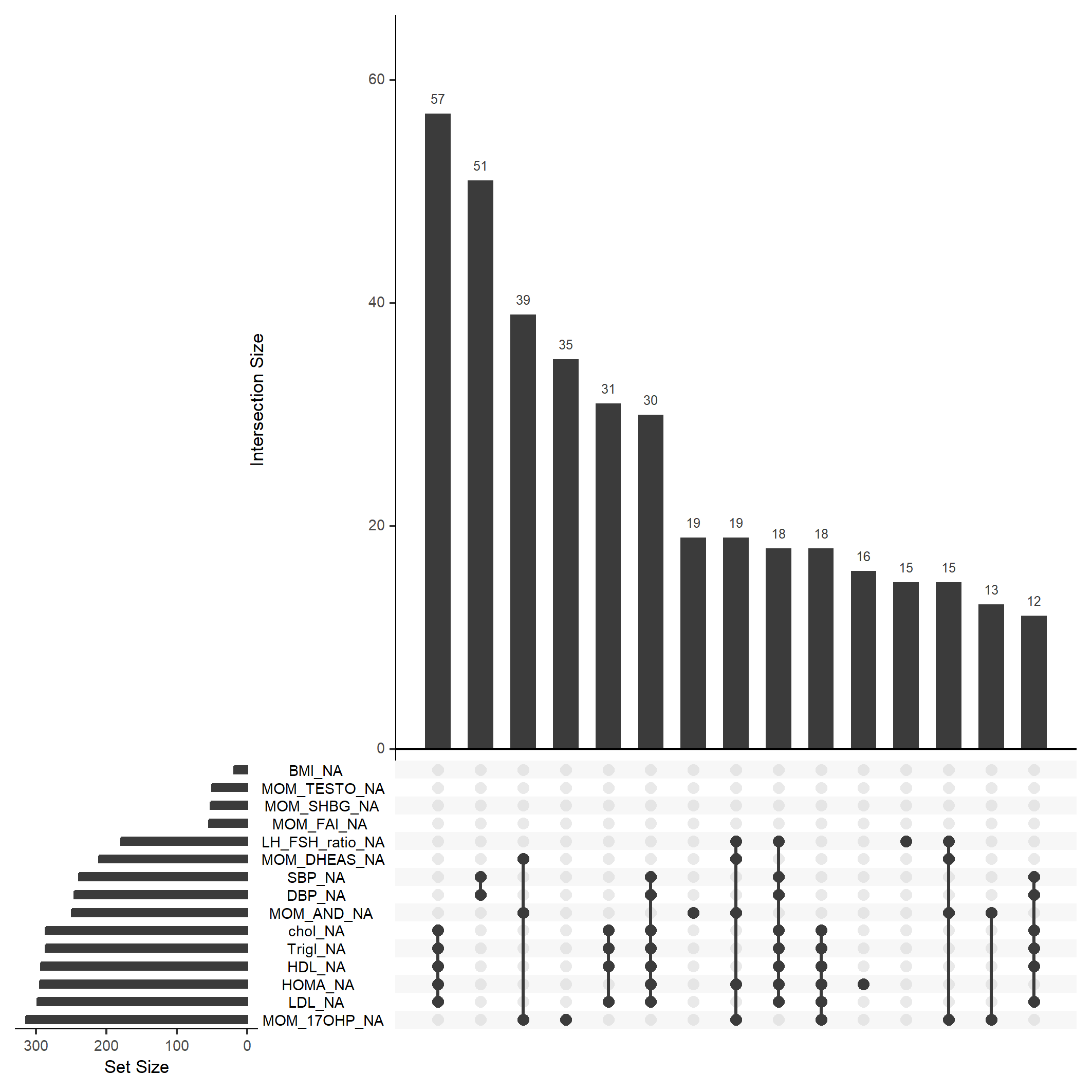

Supplement: Supplementary Figure 1 — Upset Plot for missing data. This upset plot shows the structure of missingness in the dataset. The most commonly missing variable was 17-OH-progesterone which was missing in over 300 cases. The most common set of variables jointly missing was cholesterol, HDL, LDL and HOMA which were missing together 57 times. [file Image1.tif]

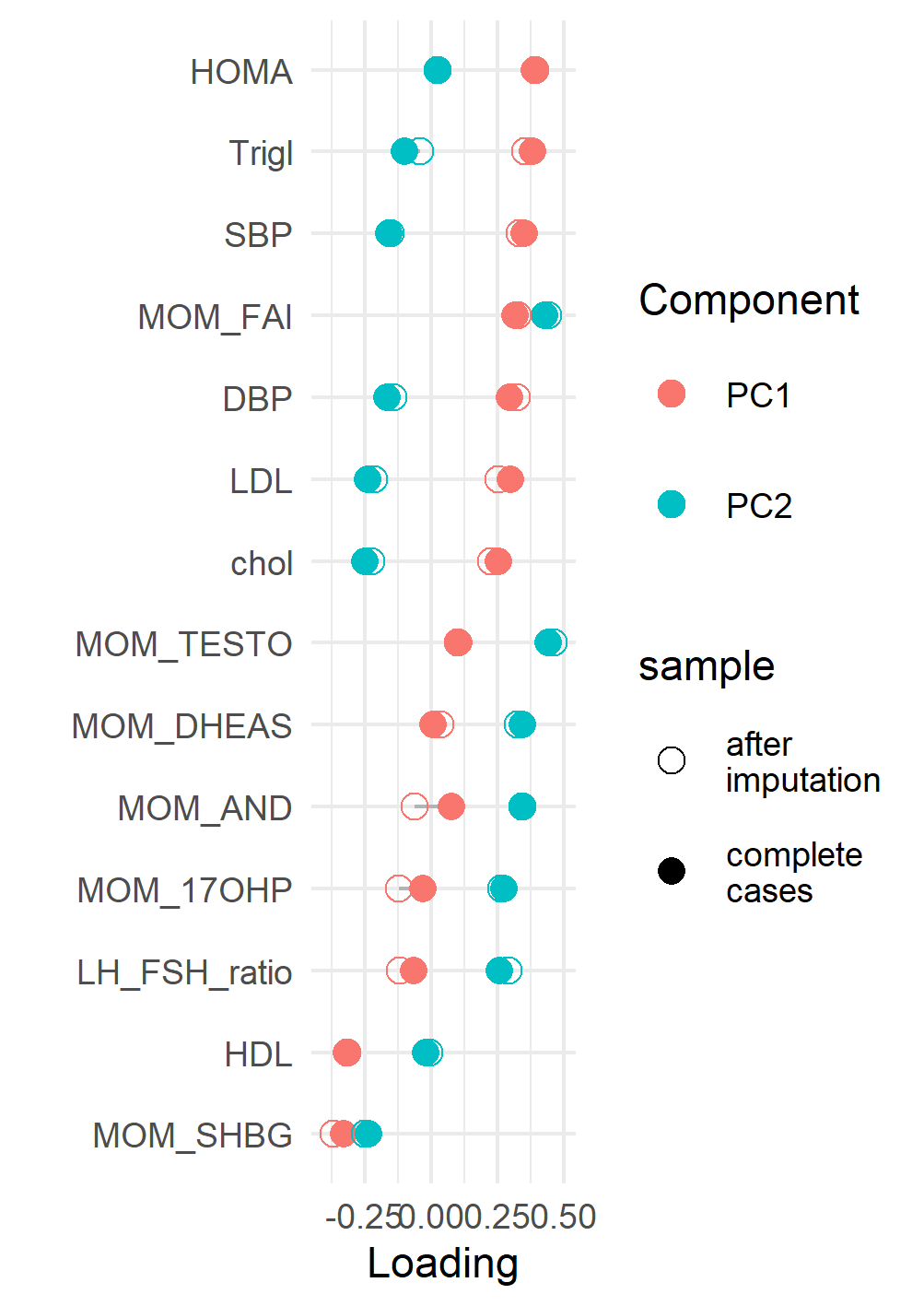

Supplement: Supplementary Figure 2 — Principal component loadings for complete-case dataset and dataset with imputation for missing data. The loadings for the first and second principal component are shown under two scenarios. The complete case scenario omits any records with missing data, leaving 294 complete records. This is compared to the full dataset of 975 with missing values imputed. The plot shows that the principal component loadings are very similar in each case. [file Image2.tif]

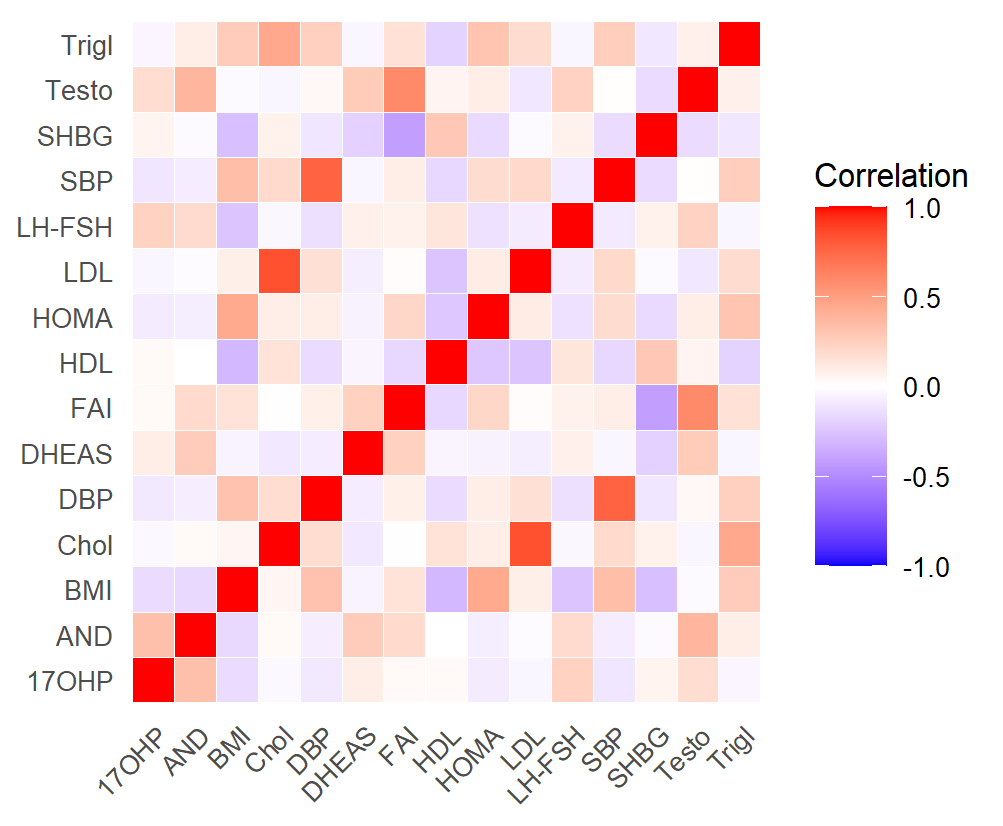

Supplement: Supplementary Figure 3 — Heatmap showing pairwise correlations between variables. Each cell represents the Pearson correlation coefficient (r) between variable pairs. Colour intensity, illustrated with the key on the right of the diagram, indicates the strength and direction of the correlation, with red indicating positive correlations and blue indicating negative correlations. Values range from –1 (strong negative correlation, blue) to +1 (strong positive correlation, red). [file Image3.tif]

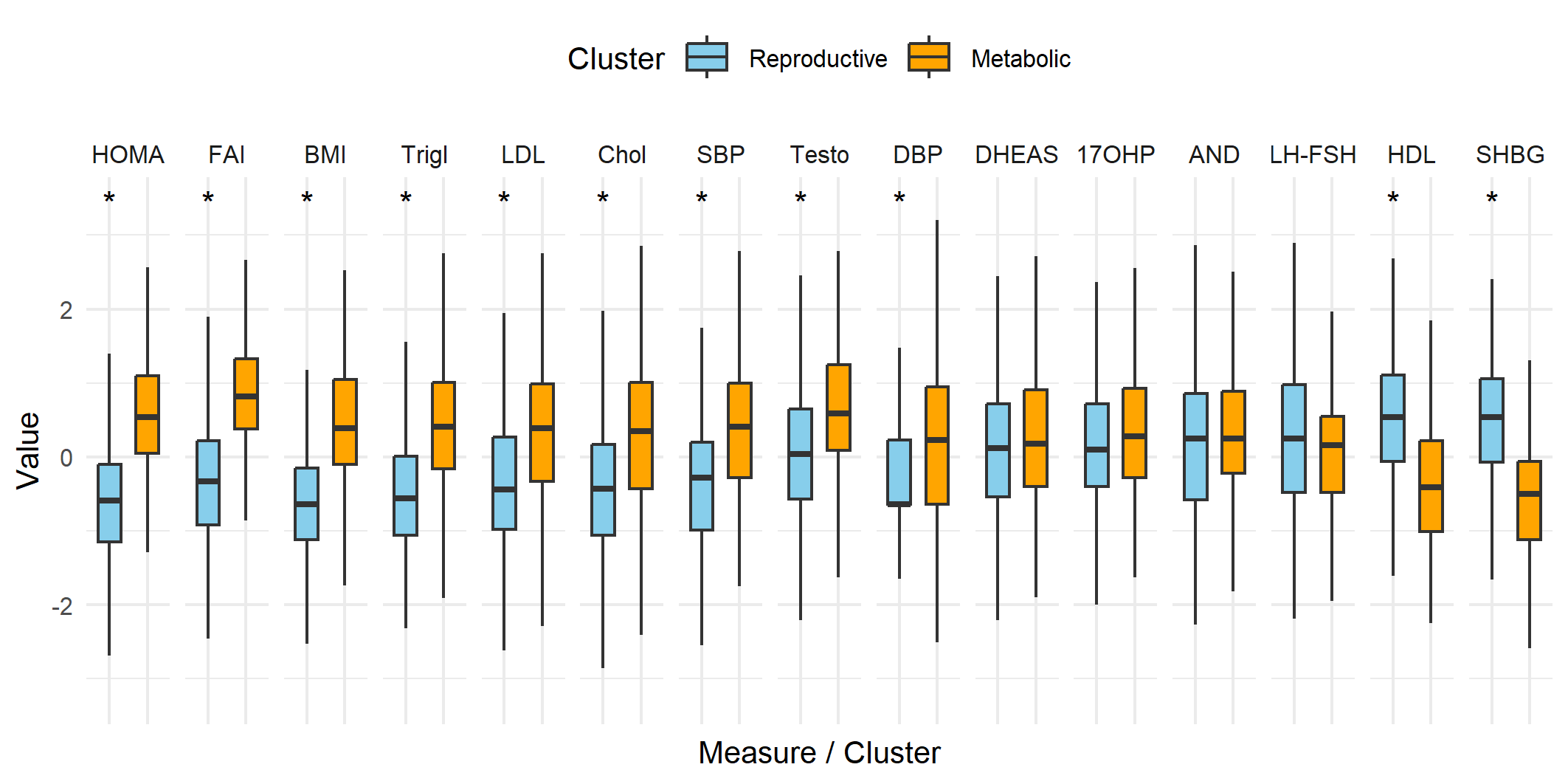

Supplement: Supplementary Figure 4 — Comparison of trait distributions between metabolic and reproductive clusters. Each of the variables were compared between Clusters A and C with evidence of significant difference in HOMA-IR, FAI, BMI, Triglycerides, LDL, cholesterol, SBP, DBP, testosterone, HDL and SHBG between groups. Significant differences in means are annotated with an asterisk, according to a two-sampled t-test with Bonferroni corrected p-values < 0.05. [file Image4.tif]

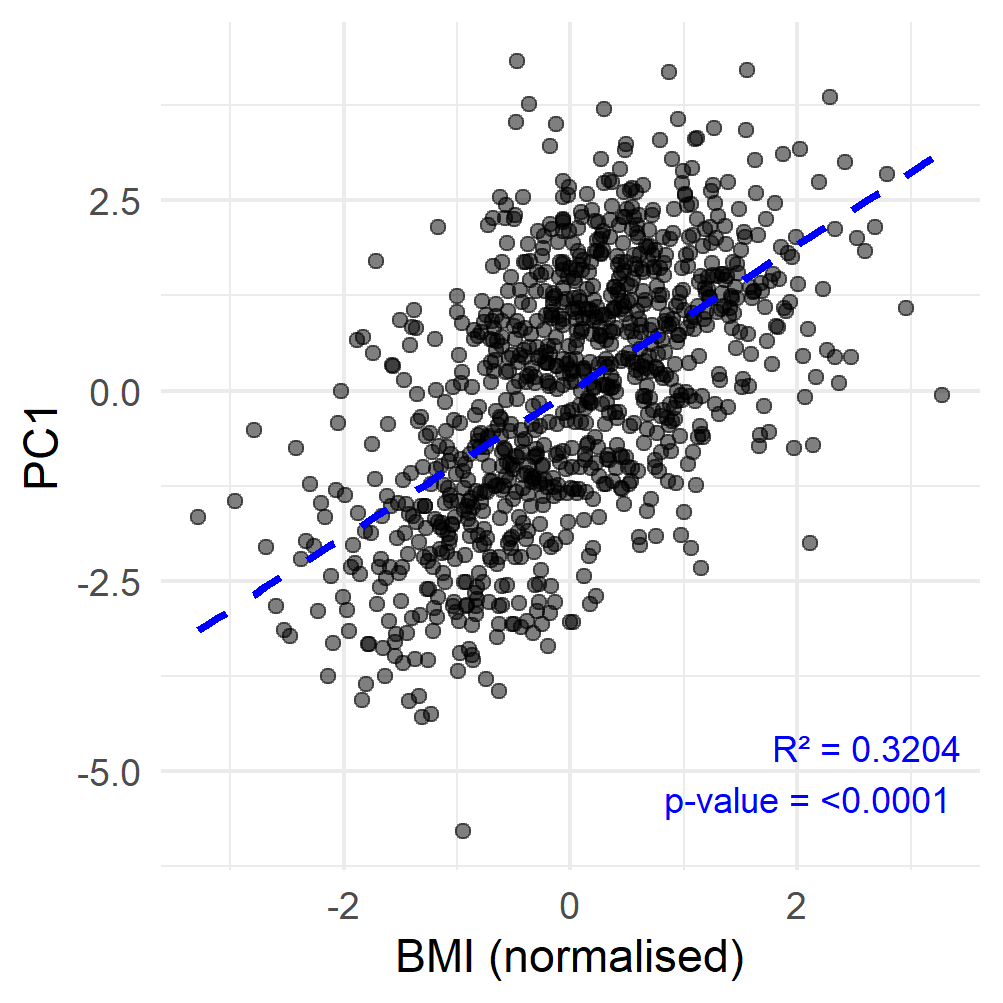

Supplement: Supplementary Figure 5 — Scatterplot of the first principal component (PC1) plotted on the X-axis against the normalised BMI score on the Y axis, with degree of correlation R2 = 0.3204, p-value <0.0001. The second component (PC2) is most strongly influenced by LH: FSH, testosterone, FAI, DHEAS and androstenedione in a similar direction, with large loadings in the opposite direction from SHBG, LDL and cholesterol. This component accounts for 18.84% of the variance in PCOS. The relationship between BMI and the second component was very weak, suggesting that BMI is less influential in this component (R2 = 0.0067,p-value 0.0107) ( Supplementary Figure S6 ). [file Image5.tif]

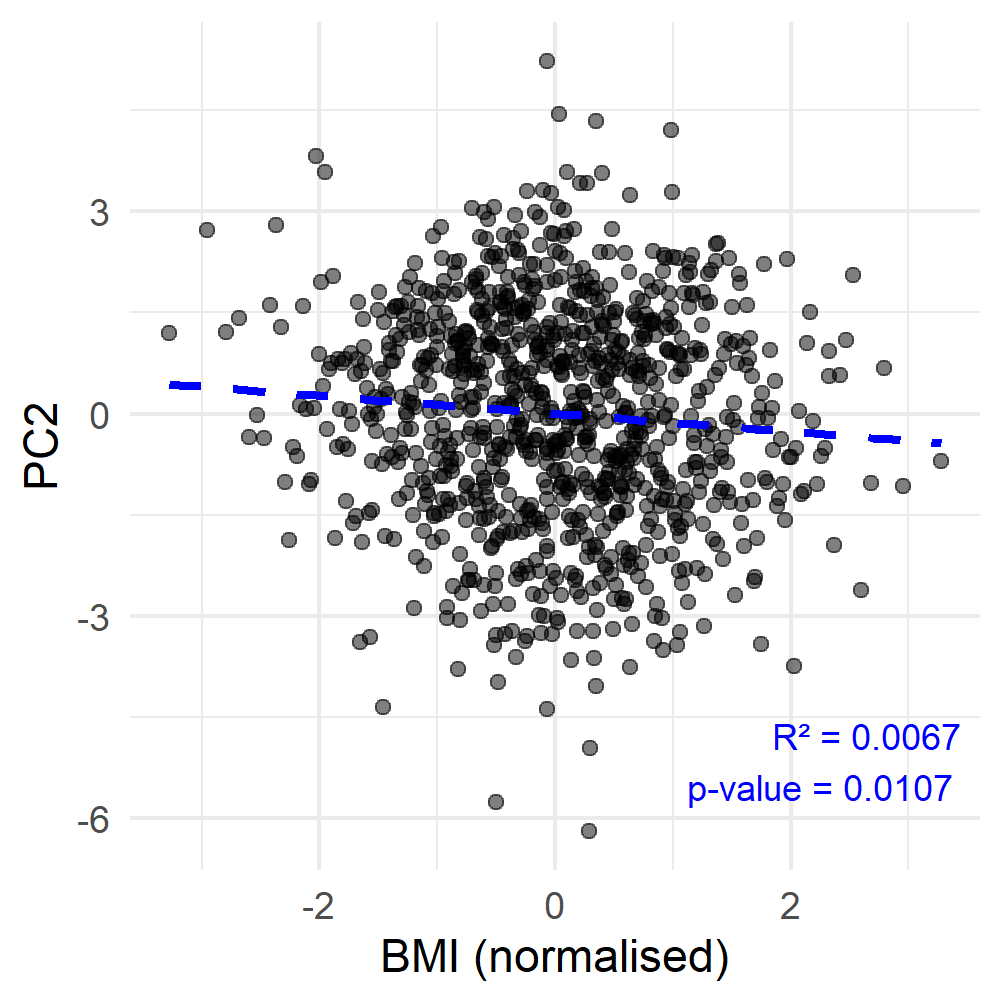

Supplement: Supplementary Figure 6 — Scatterplot of the second principal component (PC2) plotted on the X-axis against the normalised BMI score on the Y axis.Relationship between PC2 and BMI is negligible, with only very weak association demonstrated R2 = 0.0067, p-value 0.0107. [file Image6.tif]
