# Supplementary material for: Characterization of distinct polycystic ovary syndrome subtypes by cluster and principal component analyses
Source: Front Endocrinol (Lausanne). 2025 Oct 17;16:1572427. doi: 10.3389/fendo.2025.1572427 (PMC12575186; doi:10.3389/fendo.2025.1572427)
Supplement: Supplementary file 7 [file Table1.docx]

**Supplementary Table 1.** Baseline descriptive data of PCOS cases in Western Australian PCOS Cohort

| Data | n (%) | Overall  n=1035 | Lean/Normal  n=290 | Overweight  n=191 | Obese  n=533 | *P*-value |
| --- | --- | --- | --- | --- | --- | --- |
| Age at diagnosis (years) | 1035 | 28 (22.5, 34.6) | 26.1 (20.8, 30.7) | 27.7 (22.2, 35.1) | 30.0 (23.5, 36.1) | **<0.001** |
| Age of Menarche (years) | 912 | 13 (12, 14) | 13 (12, 14) | 13 (12, 14) | 12 (11, 13) | **<0.001** |
| BMI (kg/m^2^) | 1014 | 30.1 (24.6, 36.6) | 22.3 (20.2, 23.7) | 27.8 (26.3, 29.0) | 36.2 (32.9, 41.4) | **<0.001** |
| LH:FSH Ratio | 792 | 1.3 (0.9, 2.2) | 1.8 (1.1, 2.7) | 1.5 (1.0, 2.5) | 1.2 (0.8, 1.8) | **<0.001** |
| LH:FSH Ratio < 2 | 541 | 1.0 (0.7,1.4) | 1.2 (0.7, 1.5) | 1.0 (0.7,1.4) | 1.0 (0.7,1.3) | **0.025** |
| LH:FSH Ratio > 2 | 251 | 2.7 (2.2, 3.3) | 2.8 (2.3, 3.8) | 2.7 (2.3,3.1) | 2.6 (2.1, 3.0) | **0.032** |
| Fasting glucose (mmol/L) | 725 | 4.9 (4.6, 5.3) | 4.7 (4.4, 5) | 4.8 (4.5, 5.2) | 5 (4.6, 5.4) | **<0.001** |
| Fasting insulin (U/L) | 732 | 12 (7, 20) | 6 (4.2, 9) | 10 (7, 15) | 17.4 (12, 25) | **<0.001** |
| HOMA-IR | 677 | 2.7 (1.5, 4.6) | 1.3 (0.9, 1.9) | 2.2 (1.4, 3.4) | 3.9 (2.6, 5.7) | **<0.001** |
| Diabetes * n (%) | 725 | 20 (2.8) | 0 (0.0) | 2 (1.0) | 18 (3.4) | **0.005** |
| Total Cholesterol (mmol/L) | 685 | 4.8 (4.2, 5.6) | 4.7 (4, 5.3) | 4.9 (4.3, 5.7) | 4.9 (4.3, 5.6) | 0.069 |
| Triglycerides (mmol/L) | 685 | 1.1 (0.8, 1.6) | 0.7 (0.5, 1) | 1 (0.7, 1.6) | 1.3 (0.9, 1.8) | **<0.001** |
| LDL (mmol/L) | 673 | 3.0 (2.4, 3.6) | 2.8 (2.2, 3.3) | 3.1 (2.4, 3.7) | 3 (2.5, 3.7) | **0.002** |
| HDL (mmol/L) | 678 | 1.2 (1, 1.5) | 1.6 (1.3, 1.8) | 1.3 (1.1, 1.5) | 1.1 (1, 1.3) | **<0.001** |
| Testosterone | 933 | 1.8 (1.3, 2.5) | 1.9 (1.3, 2.6) | 1.7 (1.2, 2.6) | 1.8 (1.3, 2.5) | 0.862 |
| SHBG | 931 | 0.5 (0.3, 0.8) | 0.8 (0.5, 1.3) | 0.5 (0.4, 0.8) | 0.4 (0.3, 0.6) | **<0.001** |
| Free Androgen Index | 929 | 4 (2.1, 6.8) | 2.6 (1.4, 4.8) | 4.2 (1.9, 6.5) | 4.9 (2.7, 7.8) | **<0.001** |
| DHEAS | 761 | 1.2 (0.8, 1.6) | 1.2 (0.9, 1.6) | 1.2 (0.9, 1.7) | 1.15 (0.8, 1.6) | 0.283 |
| Androstenedione | 723 | 1.5 (1, 2) | 1.6 (1.2, 2.3) | 1.5 (1.1, 2) | 1.3 (0.9, 1.8) | **<0.001** |
| 17-hydroxyprogesterone | 658 | 1.6 (1, 2.5) | 1.7 (1.2, 2.5) | 1.8 (1.2, 2.8) | 1.5 (1, 2.3) | **0.009** |
| SBP (mmHg) | 756 | 112.5 (105, 125) | 110.0 (100.0, 115.0) | 110.0 (100.0, 120.0) | 120.0 (110.0, 130.0) | **<0.001** |
| DBP (mmHg) | 750 | 70 (60, 75) | 60 (60, 70) | 65 (60, 75) | 70 (60, 80) | **<0.001** |

*Diabetes – refers to proportion of patients who had ` at the time of PCOS diagnosis, based on fasting glucose or OGTT results. HOMA-IR n values are lower than fasting insulin and glucose levels, as there were some patients with fasting insulin or fasting glucose results (i.e. not collected at the same time). All parameters presented as Median (Q1, Q3), except where specified. Testosterone, SHBG, Free Androgen Index, DHEAS, androstenedione, 17-hydroxyprogesterone all presented as MOMs (multiples of the median).
